# Evaluation of Mixed Deep Neural Networks for Reverberant Speech Enhancement

**DOI:** 10.3390/biomimetics5010001

**Published:** 2019-12-20

**Authors:** Michelle Gutiérrez-Muñoz, Astryd González-Salazar, Marvin Coto-Jiménez

**Affiliations:** Escuela de Ingeniería Eléctrica, Universidad de Costa Rica, San José 11501-2060, Costa Rica; michelle.gutierrezmunoz@ucr.ac.cr (M.G.-M.); astryd.gonzalez@ucr.ac.cr (A.G.-S.)

**Keywords:** artificial neural network, deep learning, LSTM, speech processing

## Abstract

Speech signals are degraded in real-life environments, as a product of background noise or other factors. The processing of such signals for voice recognition and voice analysis systems presents important challenges. One of the conditions that make adverse quality difficult to handle in those systems is reverberation, produced by sound wave reflections that travel from the source to the microphone in multiple directions. To enhance signals in such adverse conditions, several deep learning-based methods have been proposed and proven to be effective. Recently, recurrent neural networks, especially those with long short-term memory (LSTM), have presented surprising results in tasks related to time-dependent processing of signals, such as speech. One of the most challenging aspects of LSTM networks is the high computational cost of the training procedure, which has limited extended experimentation in several cases. In this work, we present a proposal to evaluate the hybrid models of neural networks to learn different reverberation conditions without any previous information. The results show that some combinations of LSTM and perceptron layers produce good results in comparison to those from pure LSTM networks, given a fixed number of layers. The evaluation was made based on quality measurements of the signal’s spectrum, the training time of the networks, and statistical validation of results. In total, 120 artificial neural networks of eight different types were trained and compared. The results help to affirm the fact that hybrid networks represent an important solution for speech signal enhancement, given that reduction in training time is on the order of 30%, in processes that can normally take several days or weeks, depending on the amount of data. The results also present advantages in efficiency, but without a significant drop in quality.

## 1. Introduction

In real-environments, audio signals are affected by conditions such as additive noise, reverberation, and other distortions, due to elements that produce sounds simultaneously or are presented as obstacles in the signal path to the microphone. In the case of speech signals, communication devices and applications of speech technologies may be affected in their performance [1,2,3,4] by the presence of such conditions.

In recent decades, many algorithms have been developed to enhance degraded speech; these try to suppress or reduce distortions, as well as preserve or improve the quality of the perceived signal [5]. Many recent algorithms are based on deep neural networks (DNN) [6,7,8,9]. The most common implementation is based on approximating a mapping function from the degraded characteristics of speech with noise, towards the corresponding characteristics of clean speech.

The benefits of achieving this type of speech signal enhancement can be applied to signal processing in mobile phone applications, voice over Internet protocol, speech recognition systems, and devices for people with diminished hearing ability [10].

In addition to the classical perceptron model, created in the 1950s, new types of neural networks have been developed, e.g., recurrent neural networks (RNNs). An example of RNNs are the LSTM neural networks. In previous efforts to enhance speech, spectrum-derived characteristics, such as Mel-frequency cepstrum coefficients (MFCC), have been mapped successfully between noisy speech to clean speech [11,12].

The benefits of using LSTM, as well as other types of RNNs, are superior for modeling of the dependent nature of speech signals. Among the drawbacks of LSTM are the high computational cost of its training procedures.

In this work, we extend previous experiments with LSTM by evaluating deep neural networks, with a fixed number of three hidden layers, that combine LSTM layers (bidirectional) and simpler layers, based on perceptrons.

Such deep neural network algorithms have been successful in overcoming the performance of classical methods based on algorithms for signal processing, which have considered several signal-to-noise ratios (SNR) [12,13,14,15], or reverberant speech [16,17,18]. Some recent work has explored the use of mixed neural networks to achieve a better performance in different tasks, such as classifying the temporary stages of sleep, analyzing the real-time behavior of an online buyer, or the suppression of noise in a MEMS gyroscope, in which good results were obtained for specific situations and configurations [19,20,21]. The combination of different types of neural networks have been successfully presented in [22], in the form of ensemble models to predict diseases in images.

The wide variety of models applied in other fields, where regression, classification, and prediction are required, have also been analyzed [23,24], and show the multiple possibilities and the wide field of experimentation that is possible with deep neural networks.

Our main focus is on reducing the training time of the networks without a significant reduction in the capacity of the network. To achieve improvement, we consider all the different combinations of layers for de-reverberation, with the goal of accelerating the training process due to fewer connections. Thus, the process can become more efficient.

For this purpose, several objective measures were used to verify the results, which comparatively show the capacity of the BLSTM with three layers, and the combination with layers of perceptron, in improving speech conditions of reverberation. The rest of this document is organized as follows. Section 2 provides the background and context of the problem of improving reverberant speech and the BLSTM. Section 4 describes the experimental setup. Section 5 presents the results with a discussion. In Section 6, conclusions are presented.

## 2. Problem Statement

In real-world environments where speech signals are registered with microphones, the presence of reverberation is common. It is caused by the reflections of the audio signal on its path to the microphone.

This phenomenon is accentuated when the space is wide and the surfaces favor the reflection of the signals. It can be assumed that the reverberated signal *x* is a degraded version of the clean signal *s*. The relationship between both waves is described by [25]:

(1) $$x\left(n\right)=h^{⊤}\left(n\right)*s\left(n\right),$$

where $h={\left[h_{1},h_{2},\cdots ,h_{L}\right]}^{⊤}$ is the impulse response of the acoustic channel from the source to the microphone, *L* is the index of the discrete-time impulse response coefficient vector, ${}^{⊤}$ is the transpose of vector, and * is the convolution operation.

The degraded speech signal with reverberation is perceived as distant or as a very short type of echo. Consequently, this effect generally increases as the speaker’s distance to the microphone increases.

Since this effect is not desired for proper recognition and analysis of the speech signal, new algorithms have been proposed to minimize it. Mainly, in the last few years, algorithms based on deep learning have stood out.

By implementing deep neural networks, an approximation to s(n) can be estimated using a function f(·) between the data of the reverberated signal and the clean signal:

(2) $$\hat{s}\left(t\right)=f\left(x(t)\right).$$

The quality of the approximation performed by f(·) usually depends on the amount of data and the algorithm selected. For the present work, we take as a base case the estimation of f(·) made by bidirectional LSTM (BLSTM) networks with three hidden layers.

The main motivation in applying these deep neural networks is their recent success in speech enhancement related tasks, where they surpassed other algorithms applied to improve speech in noisy or reverberant conditions. In most of these experiences, it is noted the high computational cost of training the LSTM and BLSTM networks as a constraint to perform extended experimentation.

In this model, we propose a comparison and statistical validation of results with mixed networks, which include combinations of BLSTM layers and perceptron.

## 3. Autoencoders of BLSTM Networks

Since the appearance of RNNs, there are new alternatives to model the character dependent on the sequential information in applications where the nature of the parameters is relevant. These types of neural networks are capable of storing information through feedback connections between neurons in their hidden layers or another network that is in the same layer [26,27].

With the purpose of expanding the capabilities of RNNs by storing information in the short and long term, the LSTM networks shown in [28] introduce a set of gates into the memory cells capable of controlling access and storage and propagation of values across the network. The results obtained when using LSTM networks in areas that depend on previous states of information, as is the case with voice recognition, musical composition, and handwriting synthesis, were encouraging [28,29,30].

In addition to the recurring connections between the internal units, each unit in the network has additional gates for storing values: One for input, one for memory clearing, one for output, and one for activating memory. In this way, it is possible to store values for many steps or have them available at any time [28].

The gates are implemented using the following equations:

(3) $$i_{t}=\sigma \left(W_{xi}x_{t}+W_{hi}h_{t-1}+W_{ci}c_{t-1}+b_{i}\right)$$

(4) $$f_{t}=\sigma \left(W_{xf}x_{t}+W_{hf}h_{t-1}+W_{cf}c_{t-1}+b_{f}\right)$$

(5) $$c_{t}=f_{t}c_{t-1}+i_{t}\tanh\left(W_{xc}x_{t}+W_{hc}h_{t-1}+b_{c}\right)$$

(6) $$o_{t}=\sigma \left(W_{xo}x_{t}+W_{ho}h_{t-1}+W_{co}c_{t}+b_{o}\right)$$

(7) $$h_{t}=o_{t}\tanh\left(c_{t}\right)$$

where σ is the sigmoid activation function, *i* is the input gate, *f* is the memory erase gate, and $o_{t}$ is the exit gate. *c* is the activation of memory. $W_{mn}$ is the matrix that contains the values of the connections between each unit and the gates. *h* is the output of the LSTM memory unit.

Additional details about the training process and the implications of this implementation can be found at [31].

**Figure 1:**
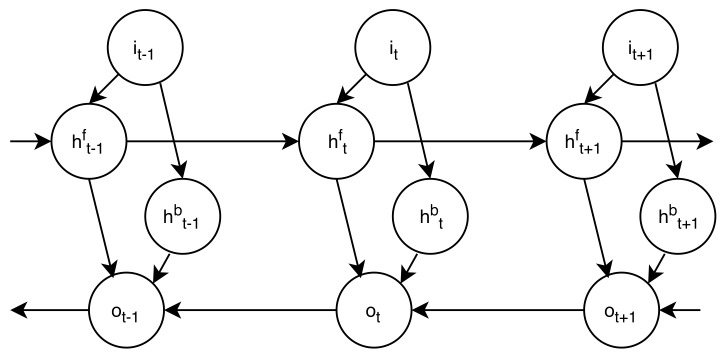
Bidirectional Long Short-term Memory (BLSTM) network structure. Adapted from [32].

An additional extension of LSTM networks that has had a greater advantage in tasks related to temporal parameter dependence is the BLSTM. Here, the configuration of the network allows the updating of parameters in both directions of the process: One can convert the input parameters to the reference of the output, and vice versa. In this work, these units are used to make comparisons. The structure of a simple bidirectional network with input *i*, output *o*, and two hidden layers ($h^{f}$ and $h^{b}$) is shown in Figure 1.

LSTM networks can handle information over long periods; however, using bidirectional LSTM (BLSTM) neural networks with two hidden layers connected to the same output layer gives them access to information in both directions. This allows bidirectional networks to take advantage of not just the past but also the future context [32].

One of the main architectures applied for regression tasks (including speech enhancement) using deep neural networks are the autoencoders. An autoencoder for speech enhancement is a neural network architecture that has been successful in various tasks related to speech [33]. This architecture consists of an encoder that transforms an input vector *s* into a representation in the hidden layers *h* through a *f* mapping. It also has a decoder that takes the hidden representation and transforms it back into a vector in the input space.

During training, the features of the distorted signal (noise or reverberation) are used as inputs for the noise elimination autoencoders, while the features of the clean speech are presented as outputs. In addition, to learn the complex relationships between these sets of features, the training algorithm adjusts the parameters of the network. Currently, computers and algorithms have the ability to process large datasets, as well as networks with several hidden layers.

## 4. Experimental Setup

To test our proposed mixed neural networks LSTM/Perceptron to enhance reverberated speech, the experiment can be summarized in the following steps:

1. Selection of conditions: Given the large number of impulse responses contemplated in the databases, we randomly chose five reverberated speech conditions. Each of the conditions has the corresponding clean version in the database.
2. Extraction of features and input-output correspondence: A set of parameters was extracted from the reverberated and clean audio files. Those of the reverberated files were used as inputs to the networks, while the corresponding clean functions were the outputs.
3. Training: During training, the weights of the networks were adjusted as the parameters with reverberation and clean were presented to the network. As usual in recurrent neural networks, the updating of the values of the internal weights was carried out using the back-propagation algorithm through time. In total, 210 expressions were used for each condition (approximately 70% of the total database) to train each case. The details and equations of the algorithm followed can be found in [34].
4. Validation: After each training step, the sum of the squared errors within the validation set of approximately 20% of the statements was calculated, and the weights of the network were updated in each improvement.
5. Test: A subset of 50 phrases, selected at random (about 10% of the total number of phrases in the database), was chosen for the test set, for each condition. These phrases were not part of the training process, to provide independence between training and testing.

In the following subsections, more details of the experimental procedure are provided.

### 4.1. Database

We used the Reverberant Voice Database created at the University of Edinburgh [35], which was designed to train and evaluate the methods of speech de-reverberation. The reverberated speech of the database was produced by convolving the recordings of 56 native English speakers with several impulse responses in various university halls. For this work, we randomly chose the following conditions: ACE Building Lobby 1, Artificial Room 1, Mardy Room 2, ACE Lecture Room 1, and ACE Meeting Room 2.

### 4.2. Feature Extraction

The pairs of WAV files corresponding to clean and reverberated speech were processed using the Ahocoder [36] software to obtain the coefficients. Those were extracted with a frame size of 160 samples and a frame-shift of 80 samples. For each frame of speech, we extracted the spectrum parameters (39 MFCC), fundamental frequency ($f_{0}$), and the energy.

For this work, neural networks were applied to improve the 39 MFCC coefficients, while the rest of the parameters remained invariant. During training, the parameters of the reverberated speech were presented as the inputs of the network, while the correspondent parameters of the clean speech were outputs.

For the test set, the MFCC parameters of the reverberated speech were substituted with the enhanced version, and the evaluation measure was applied to the reconstructed WAVE file, also made with the Ahocoder system.

### 4.3. Evaluation

For the evaluation of the results, the following objective measures were applied:

- Perceptual evaluation of speech quality (PESQ): This measure uses a model to predict the subjective quality of speech, as defined in ITU-T P.862.ITU recommendation. The results are in the range [0.5,4.5], where 4.5 corresponds to the signal enhanced perfectly. PESQ is calculated as [37]:
  (8) $$\mathrm{PESQ}=a_{0}+a_{1}D_{ind}+a_{2}A_{ind}$$
  where $D_{ind}$ is the average disturbance and $A_{ind}$ is the asymmetric perturbation. The $a_{k}$ were chosen to optimize PESQ in the measurement of general speech quality.
- Sum of squared errors (sse): This is the most common metric for the validation set error during the training process of a neural network. It is defined as:
  (9) $$\begin{array}{ccc} \mathrm{sse}(\theta ) & = & \sum_{n=1}^{T}{\left(c_{x}-\hat{c_{x}}\right)}^{2} \end{array}$$
  (10) $$\begin{array}{cc} = & \sum_{n=1}^{T}{\left(c_{x}-f\left(c_{x}\right)\right)}^{2}, \end{array}$$
  where $c_{x}$ is the known value of the outputs and $\hat{c_{x}}$ is the approximation made by the network.
- Time per epoch: This refers to the time it takes for an iteration of the training process.

Additionally, Friedman’s statistical test was used to determine the statistical significance of the results in the test sets.

### 4.4. Experiments

**Figure 2:**
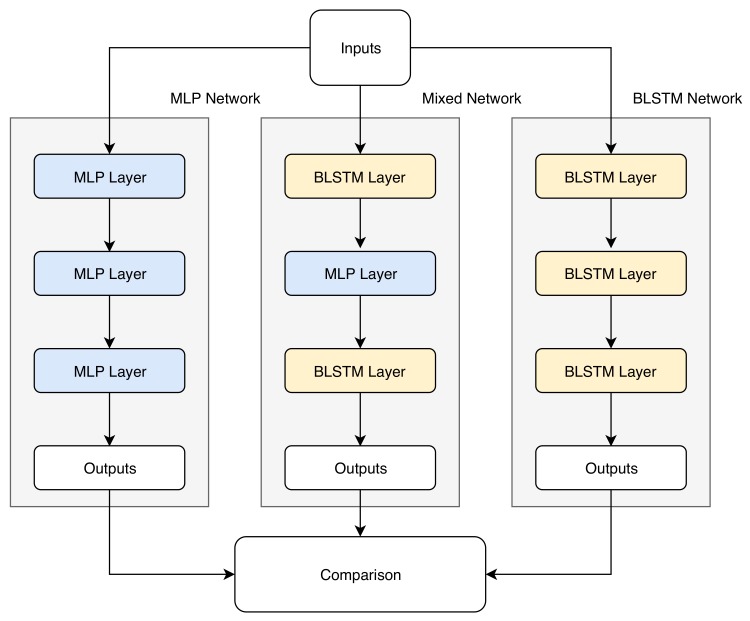
Sample of three networks compared in this work: The purely multi-layer perceptron (MPL), a mixed network, and the purely BLSTM network.

Figure 2 shows the procedure followed for the comparison between the different architectures tested in this work. To analyze all the architectures that can be formed with a mixture of BLSTM layers and MLP layers, eight different neural networks were tested for each reverberation condition:

- BLSTM–BLSTM–BLSTM
- BLSTM–BLSTM–MLP
- BLSTM–MLP–BLSTM
- BLSTM–MLP–MLP
- MLP–BLSTM–BLSTM
- MLP–BLSTM–MLP
- MLP–MLP–BLSTM
- MLP–MLP–MLP

The metrics were applied in each of these possibilities, which constitute all the possibilities that can be combined between the BLSTM and MLP layers in three layers.

## 5. Results and Discussion

Table 1 shows the training results for all networks and all possible combinations of three hidden layers. The training of each set was repeated three times, and the average values are reported. Following previously reported results, the network with only BLSTM layers provides the best results in most cases of reverberation conditions.

For the five cases of reverberation considered in this paper, the network that stands out as a competitive alternative to the three-layer BLSTM network is the MLP–BLSTM–BLSTM configuration. In addition to presenting in two cases a better result between all the architectures (under the conditions “Lecture Room” and “Meeting Room”), the training time is almost 30% less per epoch in comparison to the BLSTM network. This is one of the main indicators sought in this work.

Table 1 also shows how the training times are similar between those configurations consisting of two BLSTM layers and one MLP and those consisting of only one BLSTM layer and two MLPs. The MLP–MLP–MLP type networks, despite having very low training times per epoch, as expected, do not present competitive results in comparison to the others.

In addition to the verification of the training efficiency of the networks, Table 2 shows the results in terms of the PESQ quality metric. This is of the utmost importance, since the analysis of the problem of de-reverberation of speech signals is what is being presented. Thus, improvements in efficiency and sse values must also be checked in terms of the quality of the signal achieved.

In the last table, the differences obtained for the BLSTM–BLSTM–BLSTM base system are presented, in terms of statistical significance, according to the Friedman test.

In each of the five reverberation conditions, the results of these tests can be summarized:

- MARDY, Lecture Room and Artificial Room: Only two of the mixed configurations present results that do not significantly differ statistically with the base system. These mixed networks are BLSTM–BLSTM–MLP and MLP–BLSTM–BLSTM.
- Ace Building: In this case, three combinations of hidden layers present results that do not differ significantly from the base case.
- Meeting Room: This is a particular case, because the combination BLSTM-BLSTM-MLP is the one that presents the best result, although the improvement is not significant compared to the base system. On the other hand, MLP–BLSTM–BLSTM, BLSTM–MLP–BLSTM, and MLP–BLSTM–MLP present results that do not differ significantly from the base system.

**Figure 3:**
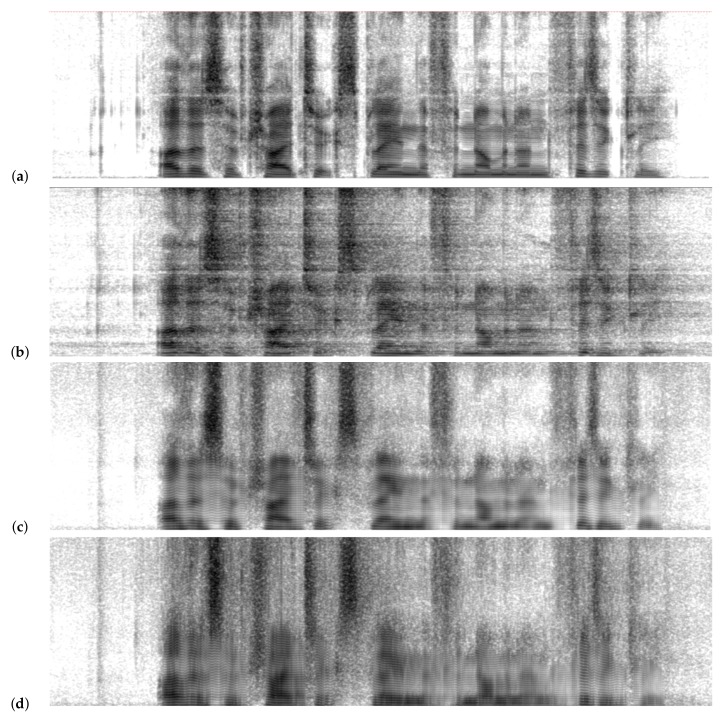
Spectrograms of a phrase in the database: (**a**) speak clean; (**b**) speak with reverberation (ACE Building Lobby); (**c**) enhancement result with the BLSTM network; and (**d**) enhancement result with the mixed MLP–BLSTM–BLSTM network.

Figure 3 shows the spectrograms corresponding to clean speech, as well as those corresponding to speech with reverberation and to two of the proposed configurations: One based solely on BLSTM layers and the mixed network that obtained better results (MLP–BLSTM–BLSTM). One can appreciate the improvements introduced by the neural networks and the proximity that is perceived visually in this representation of the spectrogram of the mixed network in comparison to that of the base system.

Considering the previous efficiency results and how these are reflected in the PESQ metric, it is emphasized that there are combinations of mixed networks, especially MLP–BLSTM–BLSTM, which reduce the times of training considerably, without significantly sacrificing the quality of results in the reverberation of the signals.However, to increase efficiency in further experiments, some processes can be parallelized and the proposal repeated in networks of greater depth.

## 6. Conclusions

In this work, the use of mixed neural networks, consisting of combinations of layers formed by perceptron units, with BLSTM layers was proposed as an alternative for the reduction of training time of purely BLSTM networks. Training time has represented a limitation for extensive experimentation with this type of artificial neural network in different applications, including some related to the improvement of speech signals.

One of the eight possible combinations of mixed networks presented competitive results, in terms of the metrics of the training system, and results that did not differ significantly from the purely BLSTM case in terms of the PESQ of the signals. The significance was determined with a statistical test. The reduction in training time is on the order of 30%, in processes that can normally take hours or days, depending on the amount of data.

The results presented here open the possibility for simplifying some neural network configurations to be able to perform extensive experimentation in different applications where it is required to map parameters with similar characteristics, as in the case of autoencoders.

## Figures and Tables

**Table 1:** Efficiency of the different combinations of hidden layers, by the condition of reverberation. * is the best value of sse in each condition.

Condition	Network (Hidden Layers)	sse	Time per Epoch (s)
MARDY	BLSTM–BLSTM–BLSTM	201.34 *	50.6
	BLSTM–BLSTM–MLP	204.39	33.3
	BLSTM–MLP–BLSTM	210.81	33.5
	BLSTM–MLP–MLP	218.91	15.9
	MLP–BLSTM–BLSTM	204.82	36.1
	MLP–BLSTM–MLP	256.32	18.6
	MLP–MLP–BLSTM	216.46	18.8
	MLP–MLP–MLP	400.34	1.2
Lecture Room	BLSTM–BLSTM–BLSTM	213.12	74.9
	BLSTM–BLSTM–MLP	214.35	48.8
	BLSTM–MLP–BLSTM	221.88	49.3
	BLSTM–MLP–MLP	229.22	23.2
	MLP–BLSTM–BLSTM	212.34 *	52.8
	MLP–BLSTM–MLP	226.39	27.7
	MLP–MLP–BLSTM	230.85	27.6
	MLP–MLP–MLP	360.41	1.8
Artificial Room	BLSTM–BLSTM–BLSTM	88.47 *	55.5
	BLSTM–BLSTM–MLP	90.37	36.5
	BLSTM–MLP–BLSTM	93.61	36.6
	BLSTM–MLP–MLP	104.23	17.4
	MLP–BLSTM–BLSTM	92.18	39.5
	MLP–BLSTM–MLP	108.56	20.6
	MLP–MLP–BLSTM	111.13	20.5
	MLP–MLP–MLP	170.61	1.3
ACE Building	BLSTM–BLSTM–BLSTM	207.32 *	73.8
	BLSTM–BLSTM–MLP	210.17	45.8
	BLSTM–MLP–BLSTM	214.29	46.1
	BLSTM–MLP–MLP	212.54	21.6
	MLP–BLSTM–BLSTM	208.04	49.2
	MLP–BLSTM–MLP	221.28	25.6
	MLP–MLP–BLSTM	220.13	25.8
	MLP–MLP–MLP	333.60	1.7
Meeting Room	BLSTM–BLSTM–BLSTM	197.37	69.9
	BLSTM–BLSTM–MLP	199.03	45.7
	BLSTM–MLP–BLSTM	204.68	45.8
	BLSTM–MLP–MLP	217.52	21.6
	MLP–BLSTM–BLSTM	196.90 *	49.6
	MLP–BLSTM–MLP	206.03	25.7
	MLP–MLP–BLSTM	214.28	25.9
	MLP–MLP–MLP	363.19	1.7

**Table 2:** Objective evaluations for the different combinations of hidden layers, by the condition of reverberation. * is the best value. The *p*-value was obtained with the Friedman test, with a significance of 0.05.

Condition	Network (Hidden Layers)	PESQ	Significative Difference	*p*-Value
MARDY	BLSTM-BLSTM-BLSTM	2.30	-	-
	BLSTM–BLSTM–MLP	2.31 *	no	0.715
	BLSTM–MLP–BLSTM	2.27	yes	0.003
	BLSTM–MLP–MLP	2.19	yes	6.648 × 10${}^{-8}$
	MLP–BLSTM–BLSTM	2.28	no	0.147
	MLP–BLSTM–MLP	2.08	yes	1.965 × 10${}^{-14}$
	MLP–MLP–BLSTM	2.24	yes	0.000
	MLP–MLP–MLP	1.94	yes	0.000
Lecture Room	BLSTM–BLSTM–BLSTM	2.28 *	-	-
	BLSTM–BLSTM–MLP	2.21	no	0.095
	BLSTM–MLP–BLSTM	2.22	yes	0.0034
	BLSTM–MLP–MLP	2.20	yes	1.729 × 10${}^{-7}$
	MLP–BLSTM–BLSTM	2.27	no	0.199
	MLP–BLSTM–MLP	2.21	yes	9.635 × 10${}^{-5}$
	MLP–MLP–BLSTM	2.20	yes	9.617
	MLP–MLP–MLP	2.00	yes	0.000
Artificial Room	BLSTM–BLSTM–BLSTM	3.18 *	-	-
	BLSTM–BLSTM–MLP	3.17	no	1.000
	BLSTM–MLP–BLSTM	3.14	yes	0.002
	BLSTM–MLP–MLP	3.12	yes	6.650 × 10${}^{-8}$
	MLP–BLSTM–BLSTM	3.17	no	1.000
	MLP–BLSTM–MLP	3.06	yes	1.965 × 10${}^{-14}$
	MLP–MLP–BLSTM	3.08	yes	2.695 × 10${}^{-6}$
	MLP–MLP–MLP	2.90	yes	0.000
ACE Building	BLSTM–BLSTM–BLSTM	2.37 *	-	-
	BLSTM–BLSTM–MLP	2.35	no	0.068
	BLSTM–MLP–BLSTM	2.35	no	0.147
	BLSTM–MLP–MLP	2.32	yes	4.22 × 10${}^{-5}$
	MLP–BLSTM–BLSTM	2.36	no	0.474
	MLP–BLSTM–MLP	2.33	yes	0.026
	MLP–MLP–BLSTM	2.33	yes	0.008
	MLP–MLP–MLP	2.08	yes	0.000
Meeting Room	BLSTM–BLSTM–BLSTM	2.28	-	-
	BLSTM–BLSTM–MLP	2.29 *	no	0.147
	BLSTM–MLP–BLSTM	2.24	no	0.060
	BLSTM–MLP–MLP	2.23	yes	0.002
	MLP–BLSTM–BLSTM	2.28	no	0.474
	MLP–BLSTM–MLP	2.25	no	0.715
	MLP–MLP–BLSTM	2.20	yes	0.001
	MLP–MLP–MLP	2.0	yes	1.960 × 10${}^{-14}$

## Abbreviations

The following abbreviations are used in this manuscript:

BLSTM	Bidirectional Long Short-term Memory Neural Network
DNN	Deep Neural Network
LSTM	Long Short-term Memory Neural Network
MEMS	Microelectromechanical System
MFCC	Mel Frequency Cepstral Coefficients
MLP	Multi-Layer Perceptron
PESQ	Perceptual Evaluation of Speech Quality
RNN	Recurrent Neural Network
SNR	Signal-to-noise Ratio
TTS	Text-to-Speech Synthesis
